# Clinico-Radiological Profile and Outcome of Airway Foreign Body Aspiration in Children: A Single-Center Experience From a Tertiary Care Center in Eastern India

**DOI:** 10.7759/cureus.22163

**Published:** 2022-02-13

**Authors:** Rekulapalli Sai Akhil, Thambi Gayathri Priya, Braja Kishore Behera, Basudev Biswal, Santosh K Swain, Debasmita Rath, Mamata Devi Mohanty, Jasashree Choudhury

**Affiliations:** 1 Pediatrics, Institute of Medical Sciences & SUM Hospital, Siksha O Anusandhan University, Bhubaneswar, IND; 2 Otolaryngology - Head and Neck Surgery, Institute of Medical Sciences & SUM Hospital, Siksha O Anusandhan University, Bhubaneswar, IND; 3 Pediatric Critical Care Medicine, Institute of Medical Sciences & SUM Hospital, Siksha O Anusandhan University, Bhubaneswar, IND

**Keywords:** bronchoscopy, aspiration, children, cough, foreign body

## Abstract

Background

Foreign body aspiration is one of the leading causes of childhood morbidity and mortality among older infants and toddler age groups. Missed and delayed diagnosis of foreign body aspiration can lead to increased incidence of complications. Early diagnosis can prevent life-threatening complications and morbidity. In this study, we aimed to evaluate the clinical and radiological details, types, localization of foreign bodies, complications, and outcomes in pediatric patients who presented to our hospital with foreign body aspiration.

Methodology

We conducted a retrospective analysis of hospital case records of children aged one month to 14 years who were admitted to the Department of Pediatrics between June 2018 and May 2020, with clinical suspicion of foreign body aspiration.

Results

A total of 22 children with a diagnosis of airway foreign body were included. The mean age of presentation was three years (SD: ±2.22), with a boy-to-girl ratio of 3.4:1. Cough (81.8%) and tachypnea (72.7%) were the most common clinical symptoms. The median duration between symptom onset and diagnosis was three (interquartile range: 6) days. Unilateral reduced breath sound (81.8%) was the most common clinical examination finding. The common site of impaction was the right main bronchus in 59.1% of cases. The foreign bodies retrieved during bronchoscopy were organic substances in 63.6% of cases, with peanuts being the most common (31.8%). Chest radiographs were normal in 36.3% of cases, and common abnormalities included hyperinflation, collapse, consolidation, and mediastinal shift. Mechanical ventilation was required in 54.5% of cases. The mean duration of hospitalization was five (SD: ±2.84) days. Complications such as pneumothorax were seen in one (4.5%) case. Mortality was seen in 4.54% of cases during the bronchoscopic procedures.

Conclusions

Foreign body aspiration was common in young male children, with cough being the common symptom. Normal X-rays of the chest were seen in one-third of cases. The common site of impaction was the right main bronchus, and organic substances such as peanuts were common foreign bodies retrieved. Strong clinical suspicion of foreign body aspiration should be kept in cases with acute onset of cough in young children. Prompt medical attention is needed to reduce the morbidity and mortality associated with foreign body aspiration.

## Introduction

Foreign body aspiration is one of the leading causes of childhood morbidity and mortality, especially among older infants and toddler age groups. According to the United States National Safety Council, mechanical suffocation accounted for 5% of accidental deaths under the age group of four [1]. As the history of foreign body aspiration may not be reliable in children, a high degree of clinical suspicion is needed for children presenting with acute onset respiratory distress. Kids have an increased risk to aspirate because of their poorly developed swallowing reflex [2]. Common foreign bodies aspirated in the airway include organic substances, such as peanuts and chickpeas, and inorganic substances, such as marbles, metallic objects, and plastic toys [2]. Chest X-ray may appear normal in a majority of laryngotracheal foreign bodies [3]. Missed and delayed diagnosis of foreign body aspiration can lead to life-threatening complications (pneumothorax, pneumomediastinum, atelectasis, laryngospasm, pneumonia, asphyxia, etc.) and morbidity [4]. Mu et al. reported that, if the diagnosis of foreign body aspiration was made within four to seven days, the complication rates were around 64% compared to 95% if it was diagnosed beyond 30 days [5]. The definite diagnosis and management of foreign body aspiration can be done by rigid bronchoscopy [1].

## Materials and methods

Study objective

In this retrospective study, we aimed to evaluate the clinical presentation and radiological details, types, localization of foreign bodies, complications during and after rigid bronchoscopy, and outcomes in pediatric patients with confirmed foreign body aspiration.

Study setting

This study was conducted in the Department of Pediatrics, Institute of Medical Sciences (IMS) & SUM Hospital, Bhubaneswar, Odisha for a period of two years from June 2018 to May 2020. The study was initiated after obtaining approval from the Institutional Ethics Committee, IMS & SUM Hospital, Bhubaneswar (IEC registration number: ECR/627/Inst/OR/2014/RR-20; approval number: Ref.no/DRI/IMS.SH/SOA/2021/081).

Inclusion and exclusion criteria

Children below 14 years of age admitted to the Department of Pediatrics with confirmed foreign body aspiration (by radiological evaluation or bronchoscopy) were included in the study. Children aged more than 14 years and those who left against medical advice were excluded from the study.

Methodology

Clinical details including demographic factors, clinical presentation, radiological features, the time interval between aspiration or symptoms to admission in the hospital, type and site of impaction, and complications associated with aspiration were collected, along with other relevant data documented as per the predefined proforma. All children received standard management (laboratory and radiological investigations, antibiotics, rigid bronchoscopy, mechanical ventilation, and other supportive care) as per the protocol during hospitalization. All children underwent rigid bronchoscopy under general anesthesia by an otorhinolaryngology surgeon. Mechanical ventilation with other supportive care was provided to unstable patients after bronchoscopic removal of foreign bodies. Stable patients with no complications during and after procedure were discharged as per clinical evaluation. Data are presented as descriptive statistics. Results were analyzed using SPSS version 20 software (IBM Corp., Armonk, NY, USA). Numerical variables are presented as frequency and percentages.

## Results

In this study, a total of 22 children with a diagnosis of airway foreign body aspiration were included. The youngest child to aspirate was one year old whereas the maximum age documented was 11 years, with a mean age of presentation of three (SD: ±2.22) years. The boy-to-girl ratio was 3.4:1 (17 boys and five girls).

A clinical history of aspiration was present only in five (22.7%) cases and was superseded by cough (81.8%) and tachypnea (72.7%). Vomiting was present in seven (31.08%) cases in our study. The median duration between symptoms and diagnosis was three (interquartile range: 6) days. Less common symptoms included noisy breathing (18.18%), hoarseness of voice (13.63%), choking (9.09%), and cyanosis (9.09%) (Table 1).

**Table 1:** Demographic and clinical presentation of the study population (n = 22). *Data are presented as number (%) unless specified otherwise. SD: standard deviation; IQR: interquartile range; PICU: pediatric intensive care unit

Demographic and clinical presentation	Number (%)*
Age in years, mean (±SD)	3 (±2.22)
Boys:girls	17:5
Duration in days between symptoms and diagnosis [Median (IQR)]	3 (6)
Cough	18 (81.8)
Tachypnea	16 (72.7)
Respiratory distress	13 (59.09)
Vomiting	7 (31.8)
SpO_2_ (Mean ± SD)	92% (±3.0)
Duration of PICU stay in days, mean (±SD)	1.36 (±2.28)
Duration of hospitalization in days, mean (±SD)	5 (±2.84)

On examination, a majority of the children had reduced breath sounds on the unilateral side (81.8%). The common site of impaction was the right main bronchus in 13 (59.1%) cases, followed by the left main bronchus in eight (36.3%) cases, and subglottic and proximal trachea in one (4.6%) case (Table 2). The foreign bodies noticed in our study (Table 3) were organic substances in 14 (63.6%) cases and non-organic substances in eight (36.4%) cases.

**Table 2:** Location of foreign body in the airway.

Site of foreign body impaction	Percentage
Right main bronchus	13 (59.1%)
Left main bronchus	8 (36.3%)
Subglottic and proximal trachea	1 (4.6%)

**Table 3:** Foreign bodies noticed in our study population.

Organic foreign bodies, 14 (63.6%)		Non-organic foreign bodies, 8 (36.4%)	
Peanuts	7 (31.8%)	Plastic toys	4 (18.1%)
Chickpeas/Yellow peas	2 (9.1%)	Marbles	1 (4.54%)
Almond nuts	1 (4.54%)	Whistles	1 (4.54%)
Tamarind seeds	1 (4.54%)	Coals	1 (4.54%)
Jackfruit seeds	1 (4.54%)	Metallic nails	1 (4.54%)
Date seeds	1 (4.54%)		
Sapota seeds	1 (4.54%)		

Chest X-rays were normal in eight (36.3%) cases. CT of the thorax was done in 18 (81.8%) cases. Hyperinflated lungs were seen in five (22.7%) cases. The collapse of the left lung was seen in three (13.6%) cases, and the collapse of the right lung was seen in two (9.1%) cases. The mediastinal shift was seen in one (4.5%) case, and consolidation was seen in one (4.5%) case. Five cases had only foreign bodies in the airway with no other associated radiological findings.

Oxygen supplementation through a face mask or nasal prongs was required in 16 (72.7%) cases during the pre-bronchoscopy period and mechanical ventilation was required in 12 (54.5%) cases in the post-bronchoscopy period. The mean duration of hospitalization was five (SD: ±2.84) days. The mean duration of pediatric intensive care admission was 1.36 (SD: ±2.28) days. Complications such as tracheobronchial injury with pneumothorax and subcutaneous emphysema were seen in one case, and slippage of foreign body from the airway to esophagus was noted in another case during bronchoscopic removal, which subsequently slipped to the esophagus and later passed in stool spontaneously during hospitalization.

Mortality was seen in one (4.54%) case during the bronchoscopic procedure where the foreign body was charcoal with delayed presentation of 45 days and was treated as non-resolving pneumonia in multiple hospitals. CT of the thorax with virtual bronchoscopy confirmed foreign body snugly fitting in the right main bronchus with difficult retrieval during bronchoscopy due to the development of granulation tissue; moreover, the child had an irreversible cardiac arrest during the procedure.

## Discussion

In this retrospective study, 72.7% of aspiration cases were seen in children under three years of age, which was similar to previously reported studies [2-4]. This can be due to the increased propensity of young children to aspirate because of their explorative nature and poorly developed swallowing reflex. The boy-to-girl ratio in this study was 3.4:1 which is comparable with the other studies reported [6-7].

The clinical history of aspiration in previous studies had a higher sensitivity (97%) and better specificity (63%) in the diagnosis of foreign body aspiration in the airway [8]. Choking or history of aspiration is a predominant clinical feature, documented in 80% of cases in different studies [8,9]. Unfortunately, this clinical clue carrying a high degree of suspicion might be missed by the care provider and later recalled retrospectively upon removal of the objects from the airway. In our study, clinical history of aspiration was present only in five (22.7%) cases, which was superseded by cough (81.8%) and tachypnoea (72.7%), which is similar to previous studies [9,10]. Vomiting was present in seven (31.08%) cases in our study. This symptom was not common in other studies and possibly present due to the posttussive effect [8-10].

The median duration between symptoms and diagnosis was three (interquartile range: 6) days, which is very high in comparison to other studies [10]. The delayed presentation may be due to less parental awareness, absence of supervision of the child, poor socioeconomic status, delayed diagnosis, and referral.

On examination, unilateral auscultation findings, such as diminished air entry, are outstanding physical signs documented in our study in 72.7% of cases, which is comparable to other studies [4].

Foreign bodies were detected in only less than one-third of cases on chest X-rays, which is higher than other studies [4]. This low sensitivity of detecting foreign bodies in the chest roentgenogram highlights that most foreign bodies consumed were organic substances that were radiolucent. Hence, a normal chest X-ray can mislead the diagnosis. Therefore, in all clinically suspicious cases, CT of the chest with virtual bronchoscopy should be done for localizing both organic and non-organic objects obstructing the respiratory tract. It can also delineate complications such as consolidation, collapse, and pneumothorax. The advantage of CT thorax has been well documented in different studies [11-13]. In our study, CT scans were done in 18 (81.8%) cases to diagnose airway foreign body aspiration.

The usual site of impaction of an aspirated object in the airway was the right mainstem bronchus. Anatomically, the right main bronchus has a larger diameter, lesser divergence angle from the axis of the trachea, and higher airflow [3,7,11]. This makes the ingress of the aspirated object more likely to go to the right mainstem bronchus. The localization of foreign bodies in the respiratory tract in our study was in the right main bronchus in 13 (59.1%) cases and the left bronchus in eight (36.3%) cases. The remaining were found to be impacted to the proximal trachea (1 (4.6%)). Similar findings were also reported by others [12-15].

The foreign bodies noticed in our study were organic substances in the majority and non-organic substances in around one-third of the cases. These findings are comparable to previous studies [4,11,12,15].

Rigid bronchoscopy-guided removal of foreign objects from the airway is the recommended standard of care in the treatment of suspected aspiration. It is the preferred method to recognize and extract the foreign object because it allows good control of the airway, visualization, and manipulation for extraction of the object from the respiratory tract [16-19]. In this study, all foreign objects were retrieved with rigid bronchoscopy and managed in the pediatric intensive care unit (PICU) after bronchoscopic removal of the foreign body. Post bronchoscopy procedure, half of the cases required invasive ventilation, which is higher in comparison to other studies due to delayed presentation [20]. The mean duration of hospitalization and the mean duration of PICU admission was slightly longer compared to other studies due to delayed presentation. The complications included pneumothorax and slippage of the foreign body from the airway to the esophagus during bronchoscopic removal.

Mortality was seen in one (4.54%) case during the bronchoscopic procedure due to cardiac arrest. The majority of the cases (72.7%) were discharged within five days of hospitalization, showing the favorable outcome of the bronchoscopic procedure [19].

## Conclusions

The diagnosis of airway foreign body aspiration becomes difficult in the absence of a history of aspiration due to reduced awareness and supervision of children by parents. Therefore, it requires a high index of suspicion for the physician to establish the possibility of foreign body aspiration. Because organic substances are more common foreign bodies to be aspirated, delayed initiation of such type of food, as well as supervising of activities and feeding of children, should be given priority. Keeping small objects away from children might be the only preventive strategy. Early diagnosis and prompt treatment aid in reducing the complications, morbidity, and mortality associated with foreign body aspiration. Future studies regarding parental awareness about foreign body aspiration should be done to address the importance of preventive strategies.
